# Toxicity of Volatile Organic Compounds Produced by Pathogens *Ewingella americana* and *Cedecea neteri* Associated with *Pleurotus pulmonarius*

**DOI:** 10.3390/toxins17090449

**Published:** 2025-09-05

**Authors:** Zhiyuan Wei, Yifan Wang, Jieheng Qiu, Yulu Nie, Lian Wang, Bin Liu

**Affiliations:** Institute of Applied Microbiology, College of Agriculture, Guangxi University, Nanning 530004, China; waizikjyun101@hotmail.com (Z.W.); wangyifan0722@126.com (Y.W.); jieheng9900@163.com (J.Q.); nieylgxu@163.com (Y.N.); wla1999@yeah.net (L.W.)

**Keywords:** *Pleurotus pulmonarius*, bacterial disease, *Ewingella americana*, *Cedecea neteri*, volatile organic compound, toxicity

## Abstract

Bacterial diseases of *Pleurotus pulmonarius*, caused by diverse pathogens and associated with a range of symptoms, reduce its commercial value and lead to substantial economic losses. While most research has focused on *Pseudomonas tolaasii* and its non-volatile toxin tolaasin, little is known about other bacterial pathogens and their volatile metabolites. In this study, two bacterial pathogens were isolated from symptomatic *P. pulmonarius* fruiting bodies in Guangxi, China, and identified as *Ewingella americana* and *Cedecea neteri*. Using headspace solid-phase microextraction coupled with gas chromatography–mass spectrometry (HS-SPME-GC-MS), we identified 16 volatile organic compounds (VOCs) produced by these two species, seven of which exhibited toxicity-inducing sunken lesions, discoloration, and inhibition of mycelial growth. Symptom severity was quantified by colorimetric analysis. Among the toxic VOCs, 2,4-di-tert-butylphenol was the most potent, inducing sunken lesions and slight discoloration at concentrations as low as 0.5 mg/mL, and causing significant inhibition of mycelial growth at 5 μg/L. The remaining VOCs also caused varying degrees of sunken lesions, yellowing or browning, and suppression of mycelial growth. This study is the first to demonstrate the pathogenic potential of VOCs produced by bacterial pathogens in *P. pulmonarius*, underscoring their role as important virulence factors and providing a foundation for further investigation into their mechanisms and control strategies.

## 1. Introduction

*Pleurotus pulmonarius* is an important edible and medicinal fungus that has attracted much attention due to its unique nutritional composition and health effects [1]. However, it is prone to disease in high-temperature and high-humidity environments, with yellow blotch and brown blotch caused by *Pseudomonas* bacteria as the major diseases. Currently, research on bacterial diseases of edible mushrooms mainly focuses on *Agaricus bisporus* [2] and *Pleurotus ostreatus* [3]. *Pseudomonas*, represented by *Pseudomonas tolaasii*, has become one of the most extensively studied pathogenic bacteria due to its widespread distribution, high pathogenicity, and diverse toxin mechanisms [4]. This bacterium can cause a series of important diseases, such as gingeri blotch disease in *A. bisporus* [5] and brown rot disease in *P. ostreatus* [6] and *Pleurotus citrinipileatus* [7]. Related research covers the molecular structure, biosynthesis pathways, mechanisms of action, and control measures of its toxin, Tolaasin. In addition, *P. fluorescens* [8], *Pseudomonas migulae* [9] and others have also been proven to cause rot or spot lesions on the fruiting bodies of various edible fungi, and *Pseudomonas* is a central topic in the study of bacterial diseases of edible fungi.

However, research on bacterial diseases of *P. pulmonarius* remains limited, particularly regarding pathogenic mechanisms, epidemiology, and control strategies. Current studies indicate that *Ewingella americana* and *Cedecea neteri* exhibit strong pathogenicity across multiple edible fungi. *C. neteri* produces phenylacetic acid and p-hydroxybenzoic acid, which induce brown spots on both mature and immature caps of *Agaricus bisporus* [10]. *C. neteri* has also been associated with yellow rot in *P. pulmonarius* [11], yellow sticky disease in *Flammulina velutipes* [12], and decay symptoms in *P. pulmonarius* [13]. *E. americana* is known to cause stipe necrosis in *Pleurotus* spp. [14] and brown rot in *Naematelia aurantialba* [15], *Lentinula edodes* [16], *Pleurotus eryngii* [17], and *F. velutipes* [18]. These infections significantly disrupt cell structure and metabolic functions, leading to reduced edibility and commercial value. Although we have successfully isolated *Ewingella americana* and *Cedecea neteri* from diseased tissues and confirmed their pathogenicity, their virulence factors, pathogenic mechanisms, and interactions with other microorganisms remain poorly understood. Currently, there is no systematic research on their transmission modes or toxic metabolites in *P. pulmonarius*, which significantly hinders both our understanding of disease complexity and the development of effective control strategies.

In the study of toxins related to bacterial diseases in edible fungi, non-volatile toxins such as lipopeptides (e.g., tolaasin) have received a great deal of attention due to their direct effects on cell membranes, their role in pore formation, and their ability to induce cell lysis [19]. Furthermore, small-molecule toxins, including monoacetylphloroglucinol (MAPG), p-hydroxybenzoic acid (PHBA), and phenylacetic acid (PAA), have also been shown to contribute to the development of mushroom brown blotch [2,10]. These toxins have therefore become important targets for the development of control measures.

However, studies into the toxicity, pathogenic mechanisms, and related effects of pathogen-derived volatile organic compounds (VOCs) on edible fungi remain limited. To date, only one study has reported that VOCs produced by *Ps. tolaasii* exhibit toxicity toward *P. ostreatus* and *P. eryngii*. Under non-sealed conditions, the VOCs produced by *Ps. tolaasii* inhibit mycelial growth in both *P. ostreatus* and *P. eryngii*, with a maximum inhibition rate of 90%. These VOCs also cause browning and softening of the fruiting body tissue of *A. bisporus* and *P. ostreatus*. GC-MS analysis revealed that *Ps. tolaasii* produces VOCs, including methanethiol, dimethyl disulfide, and 1-undecene, which are toxic to mushrooms [20]. VOCs produced by endofungal bacteria (*Pseudomonas* sp. Bi1 and De1, *Bacillus* sp. De3, *Pantoea* sp. Ma3) have been reported to exhibit antagonistic activity against *Ps. tolaasii* [21]. These compounds significantly reduced brown blotch on mushroom caps and inhibited the growth of *Ps. tolaasii* to varying degrees. Therefore, further research on the volatile toxins released by *P. pulmonarius* pathogens, particularly whether *E. americana* and *C. neteri* possess characteristic VOCs and their biological functions, will provide a new scientific basis for the rapid detection and green prevention and control of edible mushroom diseases.

Based on the above analysis, we isolated and identified *E. americana* and *C. neteri* as the primary bacterial pathogens responsible for diseases in *P. pulmonarius* cultivation facilities in Guangxi, China. In view of the current research gap concerning *P. pulmonarius* diseases, especially the limited understanding of these two atypical edible mushroom pathogens, and the significant lack of research on related toxins, particularly volatile toxins, this study aims to systematically elucidate their pathogenic features and VOC profiles. The findings are expected to provide both theoretical insights and practical guidance for elucidating the pathogenic mechanisms and virulence factors involved in bacterial diseases of *P. pulmonarius*, as well as for developing effective early warning systems and control strategies.

## 2. Results

### 2.1. Pathogen Isolation and Pathogenicity Test

**Figure 1:**
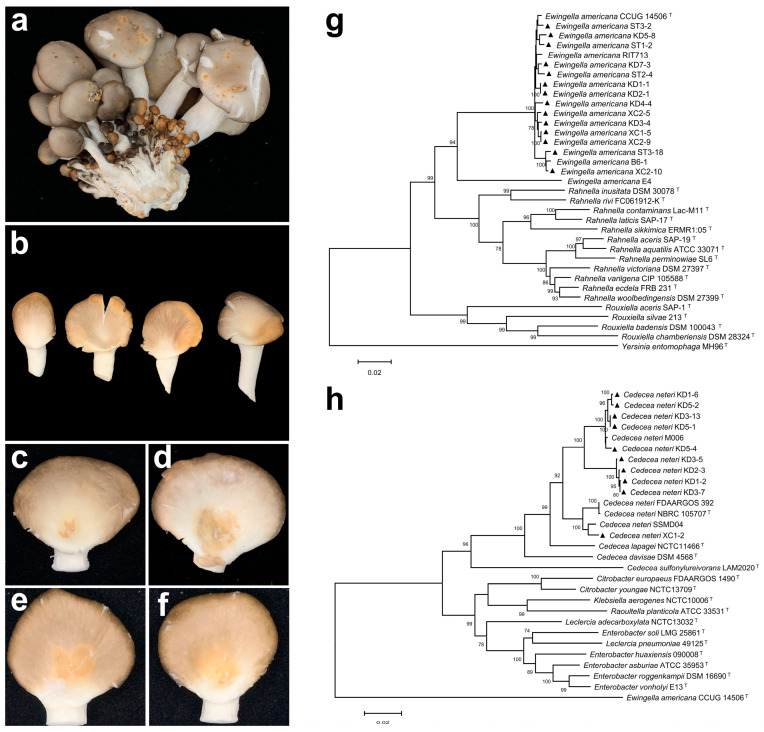
Symptoms of natural infection of blight disease and yellow rot disease in *P. pulmonarius*, pathogenicity testing, and multi-locus sequence analysis of the pathogens. (**a**) Yellow–brown spots on naturally infected fruiting bodies. (**b**) Yellow blotch on naturally infected fruiting bodies. (**c**) Symptoms 48 h after stab inoculation with *E. americana* suspension. (**d**) Symptoms 48 h after droplet inoculation with *E. americana* suspension. (**e**) Symptoms 48 h after stab inoculation with *C. neteri* suspension. (**f**) Symptoms 48 h after droplet inoculation with *C. neteri* suspension. (**g**) Phylogenetic tree of inter-species relationships within the *Yersiniaceae* family, based on concatenated sequences of 16S rRNA (1–1154 bp), *atpD* (1155–1942 bp), *dnaJ* (1943–2643 bp), *tuf* (2644–3277 bp), and *gyrB* (3278–4338 bp). (**h**) Phylogenetic tree of *C. neteri* and closely related species based on concatenated sequences of 16S rRNA (1–1333 bp), *atpD* (1334–2221 bp), *dnaJ* (2222–2933 bp), *tuf* (2934–3687 bp), and *gyrB* (3688–4777 bp). The tree was constructed using the Maximum Likelihood method (ML). ▲ indicates pathogenic strains obtained in this study. The superscript ‘T’ marks sequences from type strains.

Two distinct symptoms were observed on *P. pulmonarius* fruiting bodies collected from Nanning, Hechi, and Baise in Guangxi, China: yellow-brown spots and yellow blotches (Figure 1a,b). These symptoms resulted in a disease incidence of approximately 5–10%, significantly reducing both yield and commercial value. A total of 30 bacterial strains were isolated from symptomatic tissues, and pathogenicity tests confirmed 24 strains were pathogenic to *P. pulmonarius.* Among these, 13 pathogenic strains (KD1-1, KD1-2, KD1-6, KD2-1, KD2-3, KD3-4, KD3-5, KD3-7, KD4-4, ST1-2, ST2-4, ST3-2, ST3-18) were isolated from fruiting bodies showing yellow-brown spot, while the remaining 11 strains (KD3-13, KD5-1, KD5-2, KD5-4, KD5-8, KD7-3, XC1-2, XC1-5, XC2-5, XC2-9, XC2-10) were obtained from fruiting bodies with yellow blotch lesions.

Strains KD1-1, KD2-1, KD3-4, KD4-4, KD5-8, KD7-3, ST1-2, ST2-4, ST3-2, ST3-18, XC1-5, XC2-5, XC2-9, and XC2-10 consistently induced characteristic symptoms: stab inoculation resulted in yellow sunken spots on the caps (Figure 1c), while droplet inoculation produced expanding lesions with increasingly yellow sunken areas over time (Figure 1d). In contrast, strains KD1-2, KD1-6, KD2-3, KD3-5, KD3-7, KD3-13, KD5-1, KD5-2, KD5-4, and XC1-2 induced distinct symptoms: stab inoculation caused yellow blotches, and droplet inoculation led to yellow blotch lesions without prominent sunken areas (Figure 1f).

### 2.2. Pathogen Identification

All pathogenic strains formed ivory-colored, round, convex, smooth and opaque cultures on nutrient agar (NA) medium. These strains were Gram-negative and did not produce fluorescent pigments on King’s B medium (Figure S1).

Based on multi-locus sequence analysis (MLSA), strains KD1-1, KD2-1, KD3-4, KD4-4, KD5-8, KD7-3, ST1-2, ST2-4, ST3-2, ST3-18, XC1-5, XC2-5, XC2-9, and XC2-10 were clustered with *E. americana* (Figure 1g). All strains were catalase-positive and oxidase-negative. Esculin and salicin were hydrolyzed, whereas gelatin, arginine, and urea were not. Glucose, lactose, and mannitol were utilized, but sucrose, arabinose, and rhamnose were not. Nitrate reduction tested positive (Table S1). These characteristics are consistent with those of the *E. americana* type strain ATCC 33852 [22]. Therefore, the 14 strains were identified as *E. americana* based on MLSA, morphological traits, and physio-biochemical profiles.

The phylogenetic tree clustered the strains KD1-2, KD1-6, KD2-3, KD3-5, KD3-7, KD3-13, KD5-1, KD5-2, KD5-4 and XC1-2 with *C. neteri* (Figure 1h). These strains exhibited consistent physio-biochemical characteristics: they were catalase-positive and oxidase-negative; capable of hydrolyzing esculin, salicin, and urea, but not gelatin or arginine; utilized glucose, lactose, sucrose, mannitol, and salicin, but not arabinose or rhamnose; and tested positive for nitrate reduction (Table S2). These traits are consistent with those of the type strain *C. neteri* ATCC 33855. Based on MLSA, morphological characteristics, and physio-biochemical profiles, these 10 strains were identified as *C. neteri*.

### 2.3. Effect of VOCs from E. americana and C. neteri on P. pulmonarius

**Figure 2:**
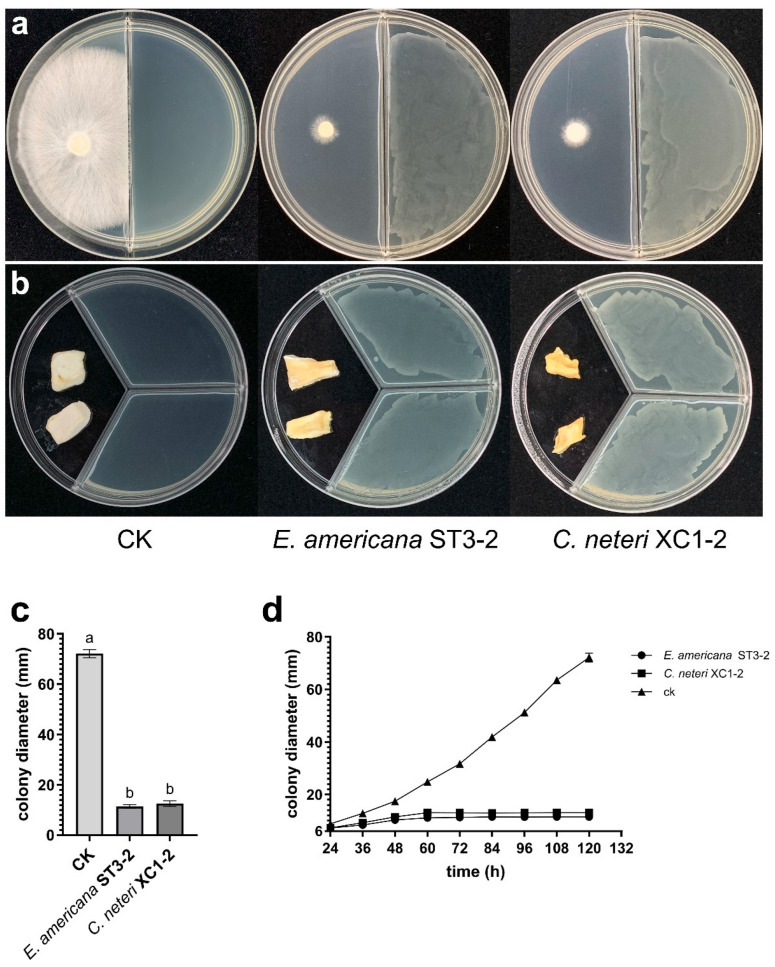
Effects of VOCs from *E. americana* and *C. neteri* on mycelial growth and tissue block morphology of *P. pulmonarius*. (**a**) Inhibition of *P. pulmonarius* mycelial growth by VOCs from two pathogenic strains after 120 h of co-culture; (**b**) Effect of VOCs from two pathogenic strains on *P. pulmonarius* tissue blocks after 72 h of exposure; (**c**) Colony diameters of *P. pulmonarius* after 120 h of exposure to VOCs. Different letters (a, b) above the bars indicate significant differences (*p* < 0.05).; (**d**) Mycelial growth curve of *P. pulmonarius* under VOCs exposure from 24 to 120 h.

In the dual-culture assay, VOCs produced by *E. americana* and *C. neteri* strains significantly inhibited the growth of *P. pulmonarius* strain TaiXiu 57 mycelium (Figure 2a,c). The mycelial growth curves (Figure 2d) showed that inhibition began at 36 h of co-culture and nearly halted growth by 60 h. These results indicate that VOCs from the two pathogenic strains reached peak inhibitory effects at 60 h, effectively suppressing the growth of *P. pulmonarius* mycelium.

The effects of VOCs produced by *E. americana* and *C. neteri* strains on *P. pulmonarius* tissue blocks were also investigated. After 72 h of exposure, VOCs from both pathogenic strains induced yellow rot symptoms in the tissue blocks, accompanied by a foul odor (Figure 2b). Among the two pathogens, *C. neteri* caused more severe symptoms, resulting in water-soaked soft rot. In contrast, the control tissue blocks showed no discoloration, and mycelial growth was observed on surface.

### 2.4. GC-MS Analysis of VOCs from E. americana and C. neteri on P. pulmonarius

As shown in Table 1, *E. americana* ST3-2 produced 9 VOCs, classified into four categories: alcohols (isoamyl alcohol, 2-ethylhexan-1-ol, 2-phenylethanol, and 2-undecanol), esters (isoamyl acetate), ketones (2-heptanone, 2-nonanone, and 2-undecanone), and phenols (2,4-di-tert-butylphenol). The three most abundant compounds were 2-nonanone (14.72%), 2,4-di-tert-butylphenol (11.93%), and isoamyl acetate (10.50%).

*C. neteri* XC1-2 produced 15 VOCs, including six ketones (2-heptanone, 2-nonanone, 2-decanone, 2-undecanone, 2-dodecanone, and 2-tridecanone), five alcohols (2-ethylhexan-1-ol, 2-heptanol, 2-nonanol, 2-phenylethanol, and 2-undecanol), one ester (isoamyl acetate), one phenol (2,4-di-tert-butylphenol), one sulfur compound (dimethyl trisulfide), and one aromatic compound (1,3-di-tert-butylbenzene). The predominant VOC was 2-undecanone, accounting for 32.71% of the total. Other compounds, such as dimethyl trisulfide, 1,3-di-tert-butylbenzene, 2-ethylhexan-1-ol, 2-heptanol and 2-dodecanone, were present at levels below 1%.

The total ion chromatograms (TICs) and mass spectrum of the GC-MS analyses are provided in Figures S2–S4.

### 2.5. Toxicity of VOCs on P. pulmonarius

**Figure 3:**
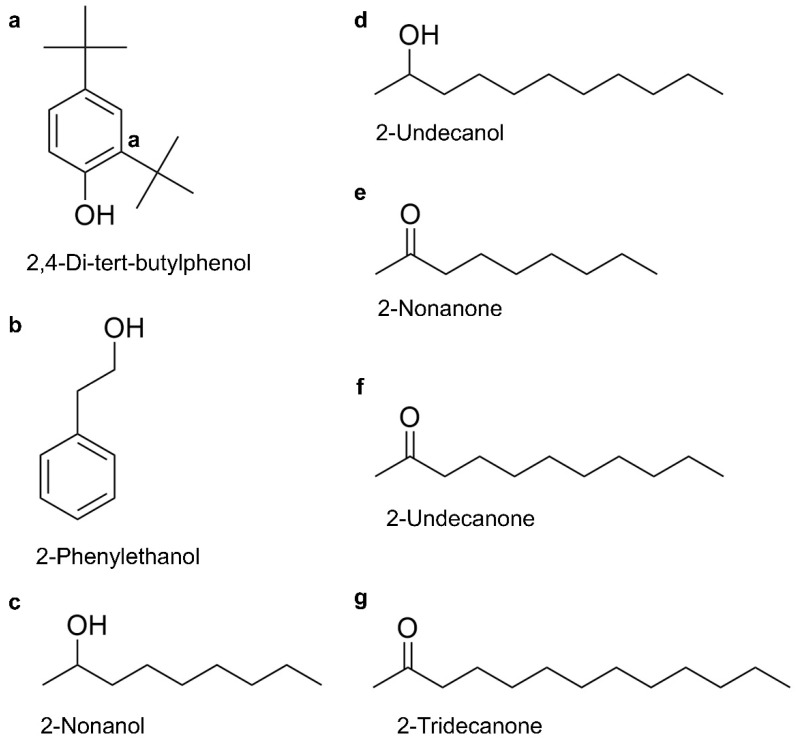
The chemical structures of seven toxic VOCs produced by *E. americana* and *C. neteri*; (**a**) 2,4-di-tert-butylphenol; (**b**) 2-phenylethanol; (**c**) 2-nonanol; (**d**) 2-undecanol; (**e**) 2-nonanone; (**f**) 2-undecanone; (**g**) 2-tridecanone.

From the 16 identified VOCs, seven compounds were selected based on their ability to induce severe discoloration in *P. pulmonarius* fruiting bodies at 4 mg/mL (Figure S5). The chemical structures of these VOCs are presented in Figure 3a–g. The toxicity of VOCs produced by *C. neteri* and *E. americana* was then evaluated on *P. pulmonarius* fruiting body and mycelial growth. Among these seven VOCs, significant differences in phytotoxic effects and concentration-dependent symptoms were observed.

**Figure 4:**
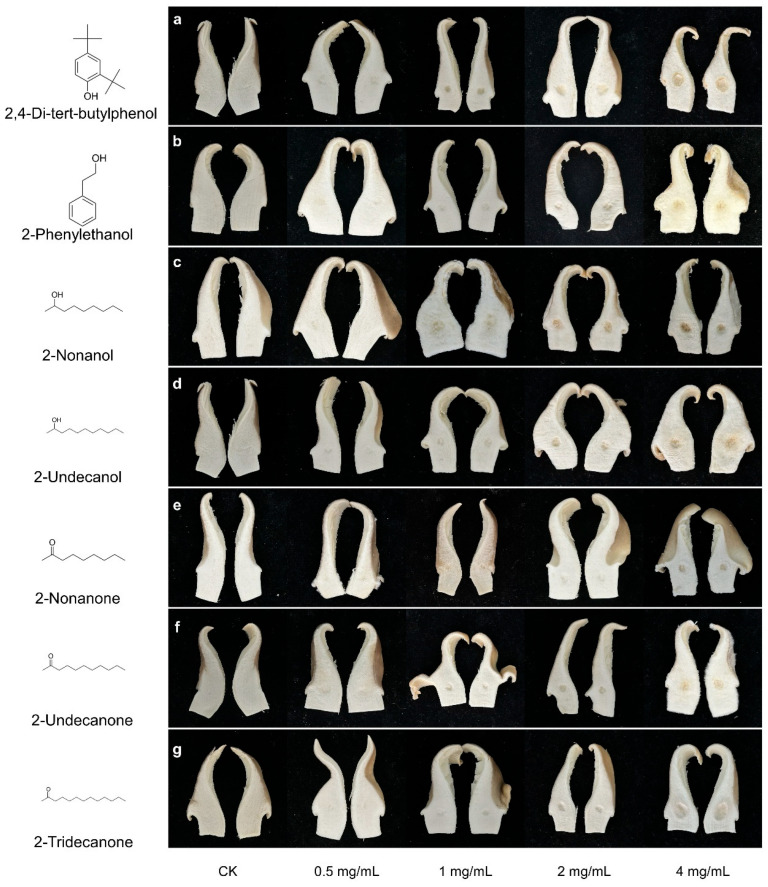
Symptoms of *P. pulnomarius* induced by different concentration of seven toxic VOCs. (**a**) 2,4-di-tert-butylphenol; (**b**) 2-phenylethanol; (**c**) 2-nonanol; (**d**) 2-undecanol; (**e**) 2-nonanone; (**f**) 2-undecanone; (**g**) 2-tridecanone. The control was a sterile 4% dimethyl sulfoxide aqueous solution used to dissolve the test compounds.

All tested VOCs induced varying degrees of tissue sinking and discoloration on *P. pulmonarius*, with severity increasing at higher concentrations (Figure 4a–g). 2,4-DTBP exhibited moderate toxicity even at low concentrations. At 0.5 mg/mL, it induced apparent sunken lesions rather than discoloration, with both symptoms worsening as the concentration increased. At 4 mg/mL, 2-tridecanone caused large crater-like sunken lesions with slight browning. Both 2-undecanone and 2-nonanone induced sunken and discoloration at concentrations ≥ 2 mg/mL. In particular, 2-undecanone caused increasingly severe browning at the inoculation site with rising concentrations, whereas 2-nonanone only induced noticeable discoloration at the highest concentration. Likewise, 2-phenylethanol caused only mild discoloration at 1–2 mg/mL but led to significant yellowing at 4 mg/mL. For 2-undecanol and 2-nonanol, mild symptoms were observed at 0.5 mg/mL. However, the toxicity of 2-undecanol increased with concentration, causing noticeable yellowing at 1 mg/mL. At 4 mg/mL, both alcohols induced pronounced discoloration and sunken lesions.

**Figure 5:**
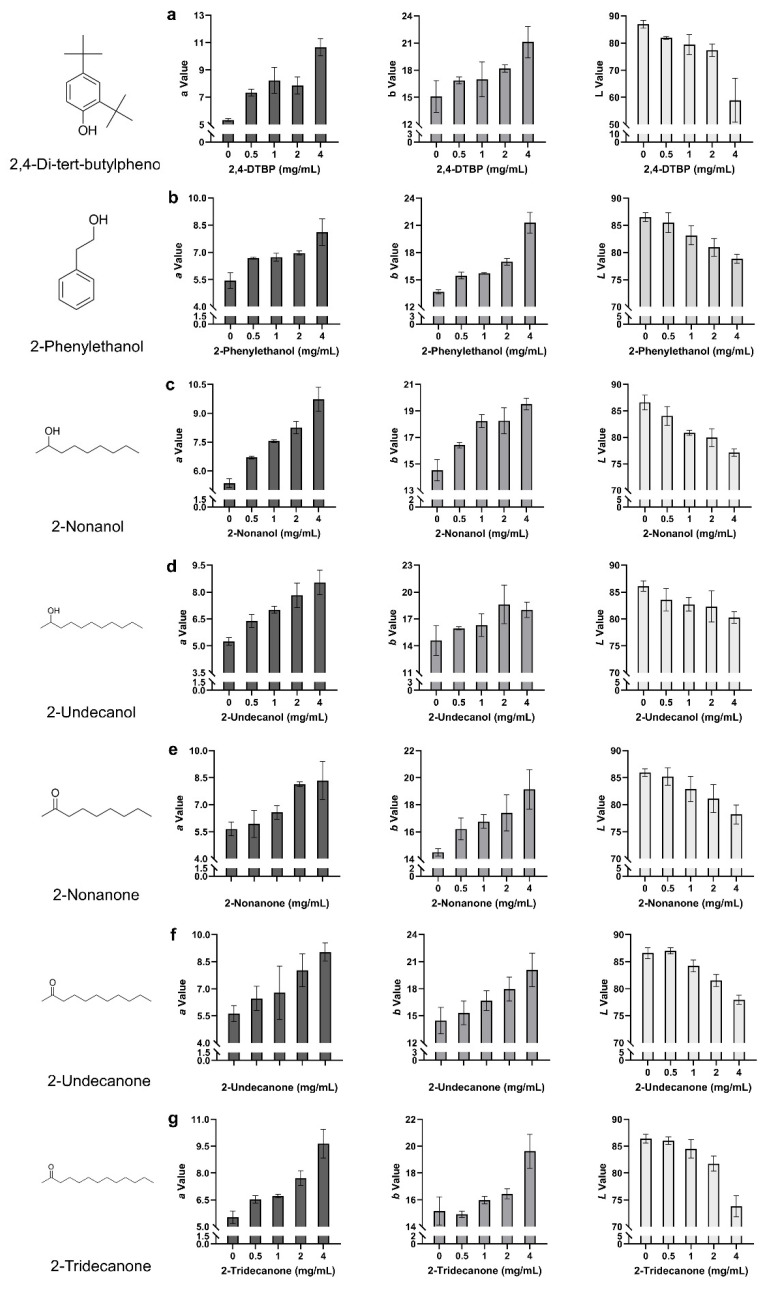
Colorimetric changes in *P. pulmonarius* fruiting bodies following topical application of seven toxic VOCs. (**a**) 2,4-di-tert-butylphenol; (**b**) 2-phenylethanol; (**c**) 2-nonanol; (**d**) 2-undecanol; (**e**) 2-nonanone; (**f**) 2-undecanone; (**g**) 2-tridecanone. Each panel shows the changes in *L* (lightness), *a* (red–green), and *b* (yellow–blue) values in response to individual VOC treatment. Threshold-dependent responses, with minimal changes at low concentrations but sharp increases in *a* and *b* at 4 mg/mL, suggesting critical concentration-triggered discoloration. Additionally, 2-tridecanone induced a steady loss of lightness (~3.5% per concentration step) and a consistent rise in *a* (+74.32%, +4.11), while *b* increased moderately (+29.42%, +4.46), indicating a predominantly browning effect. Lastly, 2-nonanol showed a distinct red-dominant profile (*a*: +62.92%, +3.30 at 4 mg/mL) with a relatively muted *b* response, suggesting suppression of yellowing pathways at high concentrations. These chromatic trends were consistent with the visible symptoms on fruiting bodies.

To quantify discoloration patterns in fruiting bodies, colorimetric analysis was conducted across the full concentration range (0–4 mg/mL). The seven VOCs induced distinct, concentration-dependent color changes in *P. pulmonarius* (Figure 5a–g). 2,4-DTBP triggered early and broad-spectrum discoloration, characterized by strong increases in redness (*a*: +99.69%, +5.32) and yellowness (*b*: +39.88%, +6.02), along with the most pronounced reduction in lightness (*L*: −32.20%, −27.98), indicating intense browning. In contrast, 2-phenylethanol produced a gradual yellowing trend, with *b* values rising from +13.19% (+1.80) at 0.5 mg/mL to +56.04% (+7.65) at 4 mg/mL, exceeding its redshift (*a*: +49.54%, +2.69). The modest decrease in *L* (−8.87%, −7.67) supports yellowing as the dominant effect. Similarly, 2-undecanol acted as a potent redshift agent, showing early and strong increases in *a* (+81.53%, +4.37 at 4 mg/mL), accompanied by moderate changes in *b* and *L*, indicative of combined reddening and browning. Both 2-nonanone and 2-undecanone exhibited.

**Figure 6:**
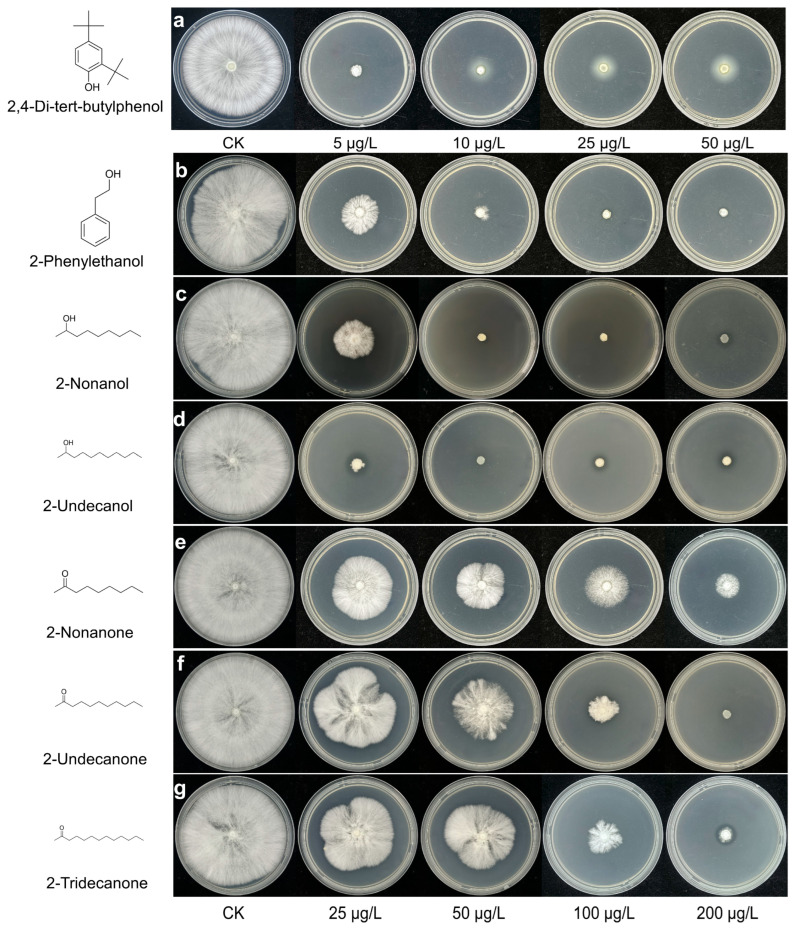
Concentration-dependent inhibition of *P. pulmonarius* mycelial growth by seven toxic VOCs. (**a**) 2,4-di-tert-butylphenol; (**b**) 2-phenylethanol; (**c**) 2-nonanol; (**d**) 2-undecanol; (**e**) 2-nonanone; (**f**) 2-undecanone; (**g**) 2-tridecanone. Each panel shows the changes in *L* (lightness), *a* (red–green), and *b* (yellow–blue) values in response to individual VOC treatment. For 2,4-DTBP, lower concentrations (5–50 μg/L) were used due to its high potency, while the other six compounds (2-undecanol, 2-nonanol, 2-phenylethanol, 2-nonanone, 2-undecanone, and 2-tridecanone) were tested at 25–200 μg/L. Inhibition was calculated relative to the untreated control. Each panel shows the concentration–response curves for each VOC. Due to its high potency, 2,4-DTBP was tested at lower concentrations (5–50 μg/L), while the other compounds were tested at 25–200 μg/L. Inhibition was calculated relative to the untreated control. Each panel shows agar plates treated with increasing concentrations of the respective VOC. A white halo appears around the central colony in panel a (2,4-DTBP) at 10, 25, and 50 μg/L, caused by condensation of 2,4-DTBP on the agar surface rather than mycelial growth. This was not observed for the other VOCs.

To further evaluate the antifungal effects of VOCs, we examined their impact on *P. pulmonarius* mycelial growth using a distinct concentration range (Figure 6a–g; Table 2). All VOCs exhibited concentration-dependent inhibition, but their efficacies varied considerably. 2,4-DTBP displayed the highest potency, causing nearly complete mycelial suppression at only 5 μg/L. Among the other compounds, 2-undecanol was the most effective, achieving 97.76% inhibition at 25 μg/L and complete suppression at higher concentrations, followed by 2-nonanol, which fully inhibited growth at 50 μg/L. 2-phenylethanol showed moderate activity, reaching complete inhibition at 100 μg/L. In contrast, 2-undecanone, 2-tridecanone, and 2-nonanone required higher concentrations for comparable effects; 2-undecanone achieved complete suppression at 200 μg/L, 2-tridecanone reached 86.88% inhibition at 100 μg/L, while 2-nonanone was the least effective, causing only 66.32% inhibition even at 100 μg/L.

## 3. Discussion

Building on our isolation and identification results, we found that *E. americana* and *C. neteri* could be simultaneously isolated from *P. pulmonarius* fruiting bodies exhibiting distinct disease symptoms. These symptoms were consistent with those previously described [11,23], suggesting a potential synergistic role of the two bacterial pathogens in disease development. *E. americana* was first identified as a pathogen of *A. bisporus* in 1995 [24], and was later reported to infect *Lentinula edodes* and *Pleurotus ostreatus* in Spain [25]. However, its association with *P. pulmonarius* has only been recognized in recent years. In contrast, *C. neteri* was not known to infect edible mushrooms until 2017, when it was found to cause disease in *Flammulina velutipes* [12]; prior to that, it had previously been known only as a human-associated pathogen. The emergence of both bacteria as *P. pulmonarius* pathogens may be linked to the rapid expansion of its cultivation, highlighting the need to clarify their pathogenic mechanisms and develop effective control strategies.

Previous studies on toxins produced by pathogens of edible fungi have primarily focused on non-volatile compounds, such as tolaasin, monoacetylphloroglucinol (MAPG), p-hydroxybenzoic acid (PHBA), and phenylacetic acid (PAA) [2,10,26]. In contrast, studies on the toxicity and pathogenicity of VOCs remain limited. VOCs produced by *Ps. tolaasii* following infection of *P. ostreatus* were reported to cause browning and decay [27]. Similarly, VOCs from *Trichoderma pleuroticola*, the pathogen of *Auricularia auricula*, were found to inhibit its mycelial growth, although specific toxicities were not identified [19]. In a broader context, VOCs have also been shown to exhibit toxicity in plant–pathogen interactions. For example, isoamyl alcohol, 2-phenylethanol, 4-ethylphenol, and 4-ethyl-2-methoxyphenol, produced by *Valsa mali*, the causal agent of apple canker, have been reported to exhibit toxicity against apple branches [28]. Although this study was conducted on plants rather than mushrooms, it nevertheless provides indirect evidence supporting the notion that VOCs from pathogens may be detrimental to their host organisms.

Compared to previously reported toxins, purified tolaasin exhibits stronger toxicity at low concentrations, inducing brown lesions on the fruiting bodies of *A. bisporus* and *P. ostreatus* at 0.06 mg/mL and 0.125 mg/mL, respectively [29,30]. MAPG, PHBA, and PAA also caused browning on *A. bisporus* at concentrations of 0.25 mg/mL, 0.5 mg/mL, and 1 mg/mL, respectively [2,10]. In contrast, 2,4-DTBP primarily caused sunken lesions on the fruiting bodies of *P. pulmonarius* at concentrations of 0.5 mg/mL or lower, with slight discoloration observed. In contrast to its effect on fruiting bodies, 2,4-DTBP exhibited stronger toxicity than tolaasin in inhibiting mycelial growth. At a concentration of 5 μg/L, 2,4-DTBP almost completely inhibited the growth of *P. pulmonarius* mycelium, while the minimum inhibitory concentrations of tolaasin for *A. bisporus* and *P. ostreatus* were 8 μg/mL and 16 μg/mL, respectively [26].

At a concentration of 4 mg/mL, 2,4-DTBP caused enlarged yellowish-brown sunken lesions at inoculation sites, which closely resembled the symptoms caused by direct inoculation with *E. americana* suspension and were similar to symptoms of *P. pulmonarius* blight disease [23]. As a lipophilic phenol identified, 2,4-DTBP exhibiting diverse biological activities such as cytotoxicity, antimicrobial effects, and phytotoxicity across a wide range of organisms [31]. It has been shown that 2,4-DTBP binds to β-tubulin of *Fusarium oxysporum*, disrupting cytoskeletal stability and inhibiting hyphal growth [32]. Transcriptomic analysis revealed that 2,4-DTBP compromises the integrity of the cell wall and membrane structures in *Ustilaginoidea virens* [33]. These findings collectively indicate that 2,4-DTBP toxicity is closely linked to the disruption of cell structural integrity, providing a molecular explanation for the formation of sunken lesions observed in *P. pulmonarius* fruiting bodies. Nevertheless, the precise molecular mechanisms of 2,4-DTBP toxicity in *P. pulmonarius* remain to be fully elucidated and warrant further investigation.

Among the other six VOCs, 2-tridecanone exhibited similar effects to 2,4-DTBP at 4 mg/mL, inducing both browning and severe sunken lesions. High concentrations of 2-nonanone and 2-undecanone also resulted in discoloration and sunken of fruiting body to varying extents. Additionally, 2-nonanol, 2-undecanol, and 2-phenylethanol caused notable yellow lesions of fruiting body at 1 mg/mL, 2 mg/mL, and 4 mg/mL, respectively. The presence of multiple VOC toxins capable of inducing discoloration suggests that these compounds may act synergistically. The term “*volatoxins*” was first coined by Bennett et al., who defined fungal *volatoxins* as volatile metabolites emitted by fungi that exert toxic effects on other organisms at physiological or moderately elevated concentrations (typically 2–10 times above ambient levels) [34]. While their definition was limited to fungal sources, the concept of *volatoxins* can be more broadly applied. In the present study, the bacterial VOCs that exhibited toxicity against *P. pulmonarius* also fulfill this criterion and can therefore be considered *volatoxins*.

Interestingly, the seven toxic VOCs identified in this study also exhibited antifungal activity against pathogenic microorganisms. For example, 2-undecanone produced by *Pythium oligandrum* caused hyphal contraction and lysis of cell membranes and organelles in *Pythium myriotylum* [35]; 2-nonanone from *Pseudomonas* sp. AN3A02 significantly reduced *Botrytis cinerea* infection rates on blueberries [36]; 2-tridecanone from *P. fluorescens* PMFe01 exhibited long-range inhibition of *Legionella pneumophila* [37]; 2-phenylethanol inhibited *B. cinerea* by disrupting cell membrane integrity, disturbing redox balance, suppressing antioxidant enzyme activity, and inducing lipid peroxidation [38]. Similar mechanisms were reported for 2-undecanol against *Rhizopus stolonifer* [39], and 2-nonanol was shown to inhibit *Alternaria solani* and *B. cinerea* [40]. These studies collectively highlight that many VOCs target cell wall/membrane structures and disrupt redox balance, consistent with the action mode of 2,4-DTBP.

In this study, we identified 16 VOCs. Both the diversity and abundance of the detected compounds were lower than those reported in other HS-SPME-GC-MS studies [41,42], and notably, no aldehydes were detected. This discrepancy may be attributed to the type of SPME fiber coating used and the polarity of the GC column. For example, a DVB/CAR/PDMS fiber combined with a polar DB-Wax column has been shown to be more effective for detecting aldehydes [43]. Additionally, factors such as fiber derivatization, GC-MS detection limits for aldehydes, and microbial differences in metabolism could contribute to the absence of aldehyde detection [44,45,46]. It is noteworthy that in our study, GC-MS chromatograms of each pathogen displayed multiple high-intensity column bleed peaks (silicon-containing peaks) that persisted throughout the analysis. GC-MS analysis of both pathogens revealed extensive column bleed peaks appearing throughout the chromatograms, potentially interfering with VOC detection. Several peaks identified as ethyl iso-allocates, which are labeled as non-microbial in origin (“Microorganism: No”) by the mVOC 4.0 platform, were still detected even after column replacement. These limitations suggest that not all VOCs produced by *E. americana* and *C. neteri* were detected, and that certain key compounds may have been obscured or interfered with by column bleed peaks. As noted [47], increased column bleed interferes with detection and causes data bias, while contamination of ion sources or optical components reduces absolute instrument sensitivity, further complicating compound identification and quantification. Therefore, future studies should consider employing more stable, low-bleed chromatographic systems and higher-resolution mass spectrometry to more accurately resolve the authentic VOC profiles produced by microorganisms.

## 4. Conclusions

In this study, we isolated and identified two bacterial pathogens of *P. pulmonarius* in Guangxi—*E. americana* and *C. neteri*—from samples showing distinct disease symptoms. Notably, this is the first report of *E. americana* as a pathogen of *P. pulmonarius* in Guangxi. Together, these two strains appear to be the major bacterial pathogens affecting *P. pulmonarius* cultivation in this region.

Using HS-SPME-GC-MS, we identified 16 volatile organic compounds (VOCs) produced by the pathogens, seven of which—including three ketones, three alcohols, and one phenolic compound—exhibited toxic effects. Among them, 2,4-DTBP showed the highest toxicity, causing significant tissue damage, discoloration, and inhibition of mycelial growth. The remaining VOCs also induced varying degrees of fruiting body discoloration and mycelial suppression. Colorimetric analysis quantitatively confirmed symptom progression, supporting the visual observations.

This study is the first to demonstrate the pathogenic potential of bacterial VOCs against *P. pulmonarius*. Our findings highlight the role of VOCs as important virulence factors, expanding understanding of mushroom-bacteria interactions and laying a foundation for future research into their mechanisms and control strategies.

## 5. Materials and Methods

### 5.1. Pathogen Isolation

To isolate the pathogen, tissues samples (5 × 5 × 5 mm) were sterilized in 75% ethanol, and triple-rinsed with sterile deionized water (SDW) (HHitech, Shanghai, China), homogenized in a microcentrifuge tube with 1 mL SDW, then serially diluted from 10^−1^ to 10^−7^. Each dilution (150 μL) was spread-plated on Luria–Bertani (LB) medium (Solarbio, Beijing, China) , and then incubated at 28 °C for 24 h. Colonies with different colors, shapes, and sizes were selected and purified two to three times on LB agar plates using streaking methods to obtain pure cultures.

### 5.2. Pathogen Analysis

Single bacterial colonies were inoculated into centrifuge tubes containing 15 mL of LB liquid medium and cultured with shaking at 150 rpm for 18 h at 28 °C. Two milliliters of the cultured bacterial suspension were transferred to a sterile EP tube, and cells were collected by centrifugation at 12,000 rpm for 1 min. The resulting cell pellet was washed and centrifuged with deionized water three times. And the final pellet was resuspended in SDW to prepare a bacterial suspension of 2 McFarland (≈6 × 10^8^ CFU/mL).

For the pathogenicity test, *P. pulmonarius* fruiting bodies with similar size and complete-looking were selected. A total of six fruiting bodies were used for each pathogen treatment, with three subjected to wound inoculation and three to droplet inoculation, each receiving 30 μL of the suspension. The inoculated mushrooms were placed in an incubator maintained at 25 °C and 80–90% relative humidity. Pathogenicity was assessed 1 to 3 days after inoculation. Bacteria were re-isolated from symptomatic tissues to compare their morphological and molecular characteristics with those of the original inoculum, thereby fulfilling Koch’s postulates.

### 5.3. Pathogenicity Identification

Genetic identification of pathogens was performed using a multi-gene approach. Genus-level classification was first achieved by sequencing the 16S rRNA gene. Subsequently, four conserved housekeeping genes were amplified using previously reported primer pairs [10,48,49,50] (Table S3). PCR was conducted in a total volume of 30 μL, comprising 15 μL of 2× Rapid Taq Master Mix, 1 μL each of forward and reverse primers, 1 μL of genomic DNA, and 12 μL of ddH_2_O. The thermal cycling program included an initial denaturation at 95 °C for 3 min; followed by 32 cycles of denaturation at 95 °C for 30 s, annealing at 44–60 °C for 30 s, and extension at 72 °C for 30 s; with a final extension step at 72 °C for 10 min. PCR products were separated on 1% agarose gel, purified, and sequenced by BGI Genomics Co., Ltd. (Shenzhen, China).

The obtained sequences were analyzed via BLAST (v2.16.0) against the NCBI GenBank database and have been deposited under the accession numbers listed in Table S2. Representative type strain sequences were retrieved from GenBank and aligned using MUSCLE implemented in MEGA X (v10.2.6). The aligned sequences were then concatenated, and a phylogenetic tree was constructed using the maximum likelihood (ML) method with 1000 bootstrap replicates.

For morphological identification, purified colonies were inoculated onto NA medium using a three-zone method and cultured for 24 h. Colony morphology and color were observed under a stereomicroscope (SMZ 745T, Nikon, Tokyo, Japan). Colonies were also inoculated into King’s B plates and observe fluorescence after 48 h. Bacterial cells were harvested into sterile EP tubes, prepared as suspensions, and Gram-stained using a commercial kit, then observed under 100× oil immersion microscopy (ECLIPSE 80*i*, Nikon, Japan).

Physiological and biochemical characterization was conducted using reagents from Hangzhou Microbial Reagent Co., (Hangzhou, China) including assays for oxidase, catalase, gelatinase, urease, arginine dihydrolase, glucose, lactose, sucrose, arabinose, rhamnose utilization, aesculin, salicin, mannitol metabolism, and nitrate reduction. Pathogens were identified by integrating morphological, physiological, biochemical, and multi-locus sequence analysis (MLSA) results.

### 5.4. Bioassay of VOCs by E. americana and C. neteri In Vitro

To evaluate the impact of pathogen-derived volatile organic compounds (VOCs) on the mycelial growth of *P. pulmonarius* strain Taixiu 57, a two-compartment petri dish confrontation assay was conducted, and growth curves were plotted. Pathogen strains were activated on LA medium and cultured in LB medium at 28 °C with shaking at 150 rpm for 12 h. The bacterial suspension was diluted with ultrapure water to 0.5 McFarland concentration (≈1.5 × 10^8^ CFU/mL). Ten milliliters of LA and PDA medium were added into separate compartment. After solidification, 100 μL of diluted bacterial suspension was spread onto the LA side and air-dried. A 6-mm mycelial block of *P. pulmonarius* (cultured for 96 h) was placed onto the PDA side. The dish was incubated at 28 °C for 120 h, and colony diameters were recorded every 12 h from 24 h using the cross-intersection method.

Bioactivity of VOCs on *P. pulmonarius* fruiting body tissue was performed using a three-compartment petri dish confrontation method [20]. 6 mL of LA medium were poured into two compartments. After air-drying, 60 μL of 0.5 McFarland bacterial suspension was applied on these compartments evenly. Internal tissue blocks excised from surface-sterilized *P. pulmonarius* fruitbodies were placed in the medium-free compartment. The dish was incubated at 28 °C for 72 h, and changes in the tissue blocks were observed.

### 5.5. HS-SPME-GC-MS Analysis of VOCs

Pathogenic bacterial strains were inoculated into flasks containing 250 mL of LB liquid medium and shaken for 60 h and equilibrated at 300 rpm and 35 °C for 30 min. Meanwhile, an SPME fiber that coated with DVB/CAR/PDMS (57328-U, Supelco, St. Louis, MO, USA), was conditioned at 270 °C for 30 min in the injection port of a gas chromatograph. The fiber was then inserted into the flask headspace for VOC extraction for 30 min under stirring.

The fiber was desorbed for 5 min in the GC injection port at 250 °C in splitless mode. Helium served as the carrier gas at 1 mL/min. GC-MS analysis was conducted on a GC-MS system (TSQ9000, Thermo, Waltham, MA, USA) with a TG-5SliMS column (Thermo, USA). The GC-MS temperature programs were optimized for *E. americana* and *C. neteri*, respectively. For *E. americana*, 35 °C for 5 min, ramped at 8 °C/min to 180 °C, then 20 °C/min to 250 °C, held for 5 min; for *C. neteri*, 40 °C for 5 min, ramped at 6 °C/min to 120 °C, then 12 °C/min to 200 °C, and 20 °C/min to 260 °C, held for 5 min. Mass spectrometry parameters included a scan range of 30–400 *m*/*z*, 0.2 s/full scan. SPME fibers reconditioned at 270 °C for 20 min after each run.

Chromatograms were processed using Thermo Xcalibur (v4.2.47) software. Compounds were identified by matching spectra against the NIST 20 database via NIST MS Search (v2.4) and validated in mVOC 4.0 (https://bioinformatics.charite.de/mvoc/. Accessed on 12 March 2025). A compound was considered microbial VOCs if they had >50% similarity in the database and were labeled “Microorganism: yes” in mVOC 4.0 platform [51].

### 5.6. Toxicity Bioassays of Individual VOCs on P. pulmonarius

To ensure solubility consistency during dilution, all VOC standards, which were purchased from Adamas-beta (Shanghai, China), were dissolved in 50% DMSO to 100 mg/mL stock solutions, diluted to 0.25–4 mg/mL with 2% DMSO, and filtered through a 22-μm micro membrane filtration. 20 μL of each VOC concentration was applied to cut surfaces of halved *P. pulmonarius* fruitbodies and the control was treated with 20 μL of 4% DMSO solution [10]. The fruit bodies were placed in petri dishes and sealed with parafilm, then incubated at 20 °C and 80% humidity for 48 h. Color differences were measured via colorimeter to calculate the toxicity of VOCs. VOCs causing spots or depressions were selected for mycelial growth assays. PDA plates were inoculated with *P. pulmonarius* mycelial and exposed to VOCs via filter paper affixed to dish lids, with doses corresponding to 25–200 μg/L (5–50 μg/L for 2,4-DTBP). Plates were sealed and incubated at 28 °C for 120 h, with colony diameters recorded by the cross method.

### 5.7. Statistical Analysis

Statistical analysis was performed using GraphPad Prism 10.4.1 with Student’s *t*-test and ANOVA followed by Tukey’s HSD test; *p* < 0.05 was considered significant.

## Figures and Tables

**Table 1:** Volatile organic compounds (VOCs) identified from *E. americana* and *C. neteri* by GC-MS.

Compound	Formula	CAS No.	*C. neteri* XC1-2		*E. americana* ST3-2	
			Prob. (%)	RPA (%)	Prob. (%)	RPA (%)
Isoamyl acetate	C_7_H_14_O_2_	123-92-2	89.34	5.55	86.60	10.50
Isoamyl alcohol	C_5_H_12_O	123-51-3	-	-	53.90	7.18
2-heptanone	C_7_H_14_O	110-43-0	71.52	1.32	51.28	2.44
2-nonanone	C_9_H_18_O	821-55-6	82.41	15.86	76.43	14.72
2-nonanol	C_9_H_20_O	628-99-9	61.93	4.36	-	-
2-phenylethanol	C_8_H_10_O	60-12-8	81.63	2.83	80.07	7.12
2-decanone	C_10_H_20_O	693-54-9	71.32	2.16	-	-
2-undecanone	C_11_H_22_O	112-12-9	81.76	32.71	66.57	2.08
1,3-di-tert-butylbenzene	C_14_H_22_	1014-60-4	70.36	0.85	-	-
2-ethylhexan-1-ol	C_8_H_18_O	104-76-7	54.68	0.37	50.19	1.21
Dimethyl trisulfide	C_2_H_6_S_3_	3658-80-8	96.45	0.11	-	-
2-heptanol	C_7_H_16_O	543-49-7	50.96	0.20	-	-
2-undecanol	C_11_H_24_O	1653-30-1	55.60	2.42	51.36	1.44
2-dodecanone	C_12_H_24_O	6175-49-1	77.61	0.85	-	-
2-tridecanone	C_13_H_26_O	593-08-8	75.98	9.37	-	-
2,4-di-tert-butylphenol	C_14_H_22_O	96-76-4	71.59	3.86	63.06	11.93

Note: Prob. = NIST match probability. RPA = Relative peak area. “-” = no detected.

**Table 2:** Inhibition rates of *P. pulmonarius* mycelial growth by seven toxic VOCs at various concentrations.

Concentration (μg/L)	2,4-DTBP	2-Phenylethanol	2-Nonanol	2-Undecanol	2-Nonanone	2-Undecanone	2-Tridecanone
	Mean ± SD (%)	Mean ± SD (%)	Mean ± SD (%)	Mean ± SD (%)	Mean ± SD (%)	Mean ± SD (%)	Mean ± SD (%)
0	0 ± 0 a	0 ± 0 a	0 ± 0 a	0 ± 0 a	0 ± 0 a	0 ± 0 a	0 ± 0 a
5	97.82 ± 0.67 b						
10	99.88 ± 0.07 c						
25	99.92 ± 0.06 c	67.11 ± 1.47 b	70.55 ± 1.56 b	97.76 ± 1.37 b	46.32 ± 0.29 b	16.75 ± 2.98 b	27.61 ± 3.42 b
50	99.91 ± 0.02 c	87.05 ± 3.19 c	99.53 ± 0.12 c	99.27 ± 0.36 bc	54.92 ± 2.84 c	45.38 ± 3.52 c	35.52 ± 2.81 c
100		99.17 ± 0.13 d	99.88 ± 0.07 c	99.86 ± 0.06 c	66.32 ± 3.87 d	79.07 ± 1.74 d	86.88 ± 1.06 d
200		99.79 ± 0.11 d	99.88 ± 0.08 c	99.86 ± 0.09 c	88.5 ± 1.66 e	99.88 ± 0.06 e	98.24 ± 0.64 e

Note: Different lowercase letters (a–e) within the same row indicate significant differences (*p* < 0.05) according to one-way ANOVA followed by Tukey’s HSD test.

## Data Availability

The original contributions presented in this study are included in the article and Supplementary Materials. Further inquiries can be directed to the corresponding author.

## Supplementary Materials

The following supporting information can be downloaded at: https://www.mdpi.com/article/10.3390/toxins17090449/s1, Figure S1: Morphological characteristics of pathogens; Figure S2: The total ion chromatogram (TIC) obtained from GC-MS analysis of the *Ewingella americana* ST3-2; Figure S3: The total ion chromatogram (TIC) obtained from GC-MS analysis of the *Cedecea neteri* XC1-2; Figure S4: Experimental and reference mass spectra of seven selected VOCs; Figure S5: Toxicity of 16 identified VOCs in *P. pulmonarius* fruiting bodies at 4 mg/mL; Table S1: Primers for sequence amplification in the MLSA of this study; Table S2: GenBank accession numbers of the sequences amplified in this study; Table S3: Physiological and biochemical characteristic of *Ewingella americana* and *Cedecea neteri*.
