# Supplementary material for: Toxicity of Volatile Organic Compounds Produced by Pathogens Ewingella americana and Cedecea neteri Associated with Pleurotus pulmonarius
Source: Toxins (Basel). 2025 Sep 5;17(9):449. doi: 10.3390/toxins17090449 (PMC12474411; doi:10.3390/toxins17090449)
Supplement: Supplementary file 1 [file toxins-17-00449-s001.zip › Supplementary figures.pdf]

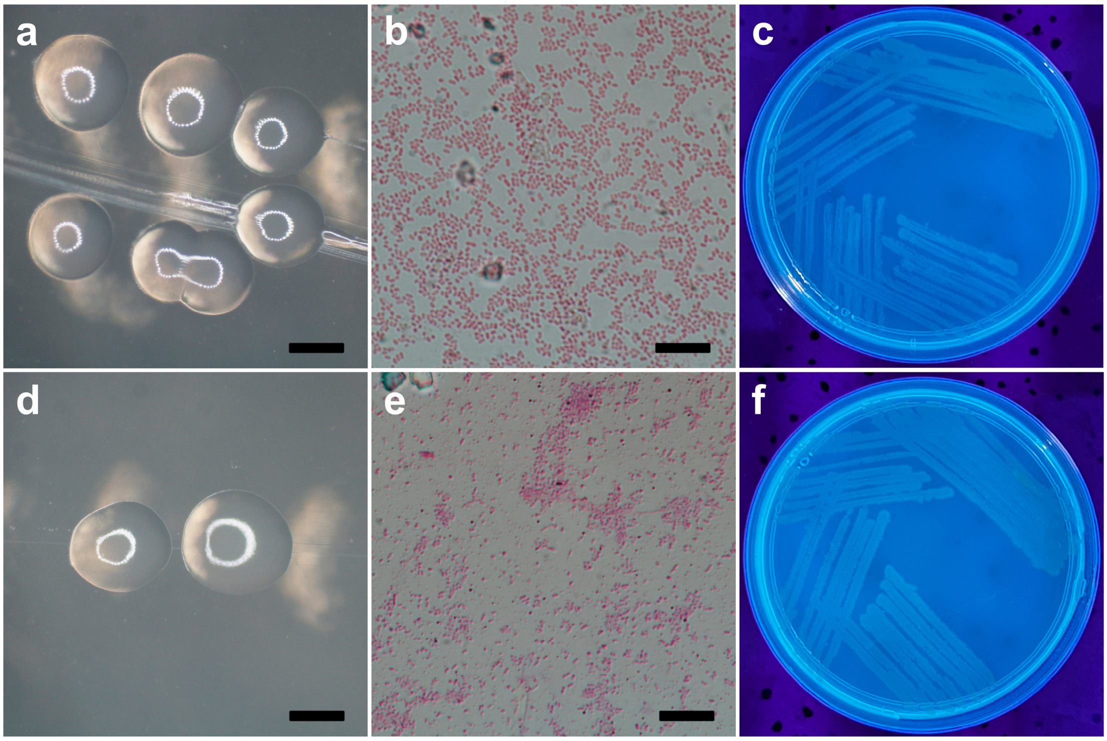

Figure S1 Morphological characteristics of pathogens.

a, d: Colony features after 24 h of cultivation on NA medim at 28 °C, Bar=200 µm. **b, e:** *E. Americana* and *C. neteri* cells of Gram-stained, Bar=10 µm**. c, f:** Characterization on King's B plates incubated at 28°C for 24 h in the dark and observed under UV light. The upper panel is *Ewingella americana* and the lower panel is *Cedecea neteri*

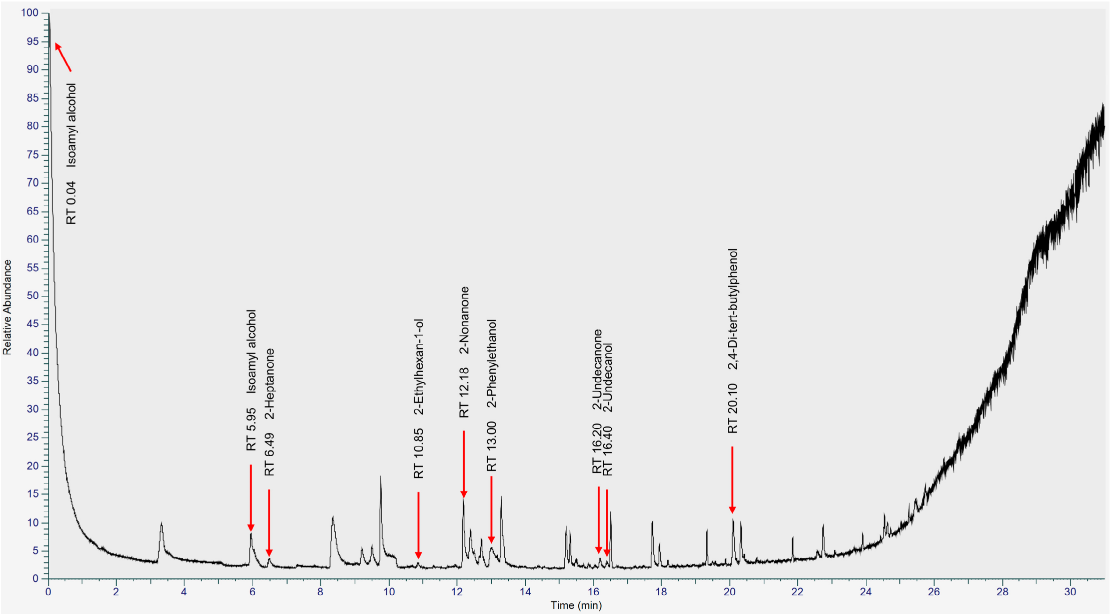

Figure S2

The total ion chromatogram (TIC) obtained from GC-MS analysis of the *Ewingella americana* ST3-2. The red arrows indicate compounds with a NIST match probability ≥ 50% and a relative peak area ≥ 0.1%. Detailed VOC profiles are provided in Appendix Tables A4

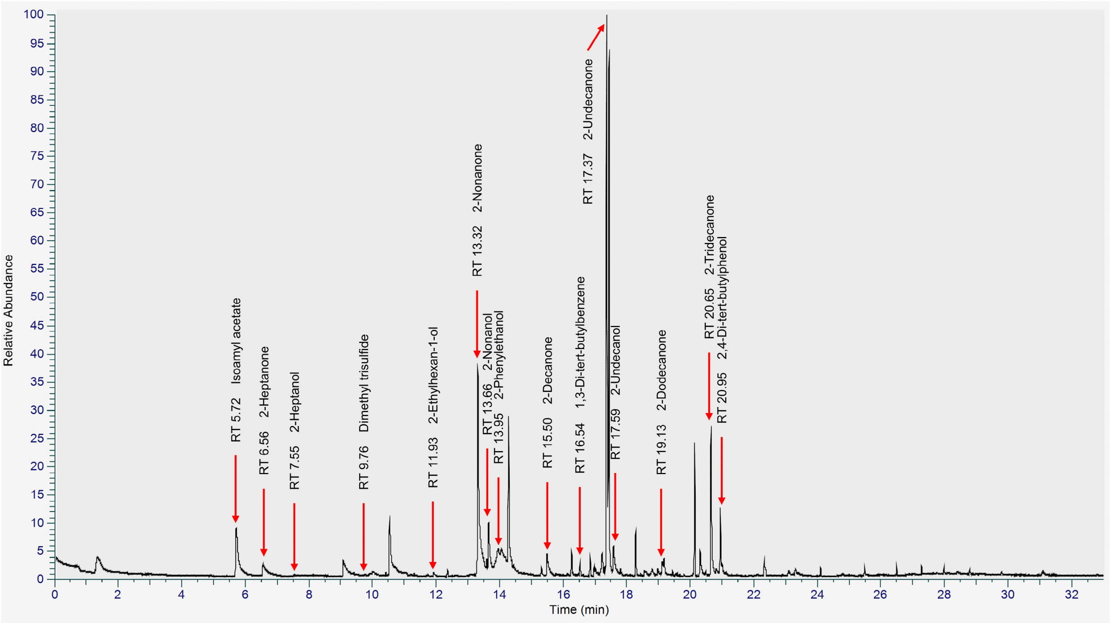

Figure S3

The total ion chromatogram (TIC) obtained from GC-MS analysis of the *Cedecea neteri* XC1-2. The red arrows indicate compounds with a NIST match probability ≥ 50% and a relative peak area ≥ 0.1%. Detailed VOC profiles are provided in Appendix Tables A5

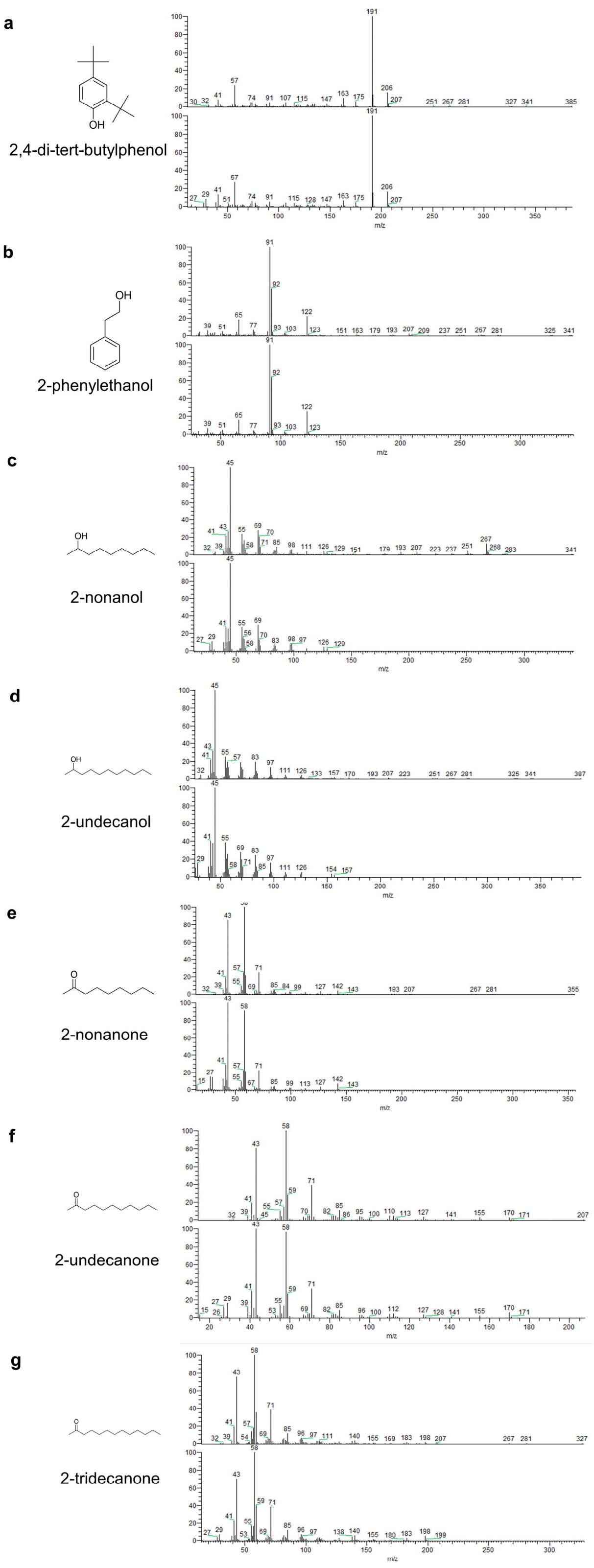

Figure S4

Comparison of the experimental and reference mass spectra of seven VOCs were selected. Upper: Experimental ESI-MS spectrum of GC-MS analysis. Lower: Reference EI-MS spectrum from the NIST 20 database. **a**: 2,4-di-tert-butylphenol; **b**: 2-phenylethanol; **c**: 2-nonanol; **d**: 2-undecanol; **e**: 2-nonanone; **f**: 2-undecanone; **g**: 2-tridecanone.

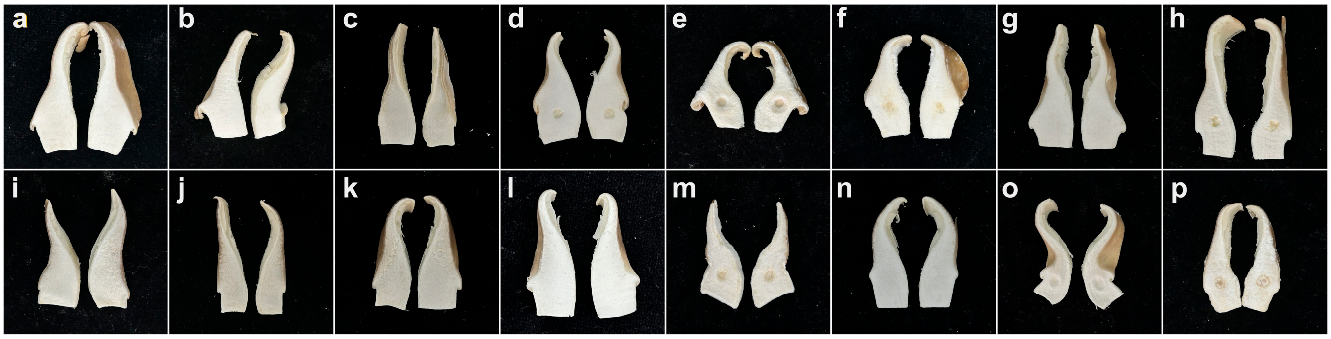

Figure S5

The seven VOCs selected from 16 identified compounds based on their toxicity in *P. pulmonarius* fruiting bodies at 4 mg/mL. **a**: Isoamyl acetate; **b**: Isoamyl alcohol; **c**: 2-heptanone; **d**: 2-nonanone; **e**: 2-nonanol; **f**: 2-phenylethanol; **g**: 2-decanone; **h**: 2-undecanone; **i**: 1,3-di-tert-butylbenzene; **j**: 2-ethylhexan-1-ol; **k**: Dimethyl trisulfide; **l**: 2-heptanol; **m**: 2-undecanol; **n**: 2-dodecanone; **o**: 2-tridecanone; **p**: 2,4-di-tert-butylphenol.
