# Supplementary material for: Toxicity of Volatile Organic Compounds Produced by Pathogens Ewingella americana and Cedecea neteri Associated with Pleurotus pulmonarius
Source: Toxins (Basel). 2025 Sep 5;17(9):449. doi: 10.3390/toxins17090449 (PMC12474411; doi:10.3390/toxins17090449)
Supplement: Supplementary file 1 [file toxins-17-00449-s001.zip › Supplementary tables.pdf]

Table S1 Primers for sequence amplification in the MLSA of this study

Primer	Target gene	Length (bp)	Tm (°C)	Primer sequence	Reference
27F	16S rRNA	20	54	AGAGTTTGATCMTGGCTCAG	Galkiewicz and Kellogg [49]
1492R		19	54	GGTTACCTTGTTACGACTT	
DN1-1F	*dnaJ*	20	51	GATYTRCGHTAYAACATGGA	Nhung et al. [50]
DN1-2R		19	55	TTCACRCCRTYDAAGAARC	
tuf-F	*Tuf*	15	48	CCCGACTCGTCACTA	Huang et al, [10]
tuf-R		15	44	GGCATTACCATCTCT	
atpD-F	*atpD*	15	42	ACAACAATGAGAAGC	Huang et al, [10]
atpD-R		15	44	ATAGACTGAACGCCA	
UP-1	*gyrB*	41	71	GAAGTCATCATGACCGTTCTGC AYGCNGGNGGNAARTTYGA	Yamamoto and Harayama [51]
UP-2r		44	75	AGCAGGGTACGGATGTGCGAGC CRTCNACRTCNGCRTCNGTCAT	

Table S2 NCBI GenBank accession numbers of the sequences amplified in this study.

Strains	Species	16s	*atpD*	*dnaJ*	*tuf*	*gyrB*
KD1-1	*Ewingella americana*	PV797003	PV808636	PV808660	PV808684	PV808708
KD2-1	*Ewingella americana*	PV797004	PV808635	PV808659	PV808683	PV808707
KD3-4	*Ewingella americana*	PV797005	PV808634	PV808658	PV808682	PV808706
KD4-4	*Ewingella americana*	PV797006	PV808633	PV808657	PV808681	PV808705
KD5-8	*Ewingella americana*	PV797007	PV808632	PV808656	PV808680	PV808704
KD7-3	*Ewingella americana*	PV797008	PV808631	PV808655	PV808679	PV808703
ST1-2	*Ewingella americana*	PV797009	PV808630	PV808654	PV808678	PV808702
ST2-4	*Ewingella americana*	PV797010	PV808629	PV808653	PV808677	PV808701
ST3-18	*Ewingella americana*	PV797011	PV808628	PV808651	PV808675	PV808699
ST3-2	*Ewingella americana*	PV797012	PV808627	PV808652	PV808676	PV808700
XC1-5	*Ewingella americana*	PV797013	PV808626	PV808650	PV808674	PV808698
XC2-10	*Ewingella americana*	PV797014	PV808623	PV808647	PV808671	PV808695
XC2-5	*Ewingella americana*	PV797015	PV808625	PV808649	PV808673	PV808697
XC2-9	*Ewingella americana*	PV797016	PV808624	PV808648	PV808672	PV808696
KD1-2	*Cedecea neteri*	PV797017	PV808637	PV808661	PV808685	PV808709
KD1-6	*Cedecea neteri*	PV797018	PV808638	PV808662	PV808686	PV808710
KD2-3	*Cedecea neteri*	PV797019	PV808639	PV808663	PV808687	PV808711
KD3-5	*Cedecea neteri*	PV797020	PV808640	PV808664	PV808688	PV808712
KD3-7	*Cedecea neteri*	PV797021	PV808641	PV808665	PV808689	PV808713
KD3-13	*Cedecea neteri*	PV797022	PV808642	PV808666	PV808690	PV808714
KD5-1	*Cedecea neteri*	PV797023	PV808643	PV808667	PV808691	PV808715
KD5-2	*Cedecea neteri*	PV797024	PV808644	PV808668	PV808692	PV808716
KD5-4	*Cedecea neteri*	PV797025	PV808645	PV808669	PV808693	PV808717
XC1-2	*Cedecea neteri*	PV797026	PV808646	PV808670	PV808694	PV808718

Table S3 Physiological and biochemical characteristic of *Ewingella americana* and *Cedecea* neteri.

Characteristic	*Ewingella americana*			*Cedecea neteri*		
	ST3-2	KD7-3	XC2-10	KD1-6	XC1-2	KD5-4
Oxidase	–	–	–	–	–	–
Catalase	+	+	+	+	+	+
Gelatin hydrolysis	–	–	–	–	+	–
Arginine dihydrolase	–	–	–	–	–	–
Urease	–	–	–	+	+	+
Glucose	+	+	+	+	+	+
Lactose	+	+	+	+	+	–
Sucrose	–	–	–	+	+	+
Arabinose	–	–	–	–	–	–
Rhamnose	–	–	+	–	–	–
Esculin	+	+	+	+	+	–
Salicin	+	+	+	+	+	+
Mannitol	+	+	+	+	+	+
Nitrate reduction	+	+	+	+	+	+

"+"= positive; "–"= negative

Reference

10. Huang, Z.X.; Huang, Y.Y.; Nie, Y.L.; Liu, B. Biological Characteristics of Two Pathogens Causing Brown Blotch in *Agaricus bisporus* and the Toxin Identification of *Cedecea neteri*. *Phytopathol. Res*. **2024**, *6*, 21. https://doi.org/10.1186/s42483-024-00239-8.
49. Galkiewicz, J.P.; Kellogg, C.A. Cross-kingdom amplification using bacteria-specific primers: Complications for studies of coral microbial ecology. *Appl. Environ. Microbiol.* **2008**, *74*, 7828–7831. https://doi.org/10.1128/AEM.01303-08.
50. Pham, H.N.; Ohkusu, K.; Mishima, N.; Noda, M.; Shah, M.M.; Sun, X.; Hayashi, M.; Ezaki, T. Phylogeny and species identification of the family Enterobacteriaceae based on *dnaJ* sequences. *Diagn. Microbiol. Infect. Dis.* **2007**, *58*, 153–161. https://doi.org/10.1016/j.diagmicrobio.2006.12.019.
51. Yamamoto, S.; Harayama, S. PCR amplification and direct sequencing of *gyrB* genes with universal primers and their application to the detection and taxonomic analysis of *Pseudomonas putida* strains. *Appl. Environ. Microbiol.* **1995**, *61*, 1104–1109. https://doi.org/10.1128/AEM.61.3.1104–1109.
