# Supplementary figures and images for: Toxicity of Volatile Organic Compounds Produced by Pathogens Ewingella americana and Cedecea neteri Associated with Pleurotus pulmonarius
Source: Toxins (Basel). 2025 Sep 5;17(9):449. doi: 10.3390/toxins17090449 (PMC12474411; doi:10.3390/toxins17090449)

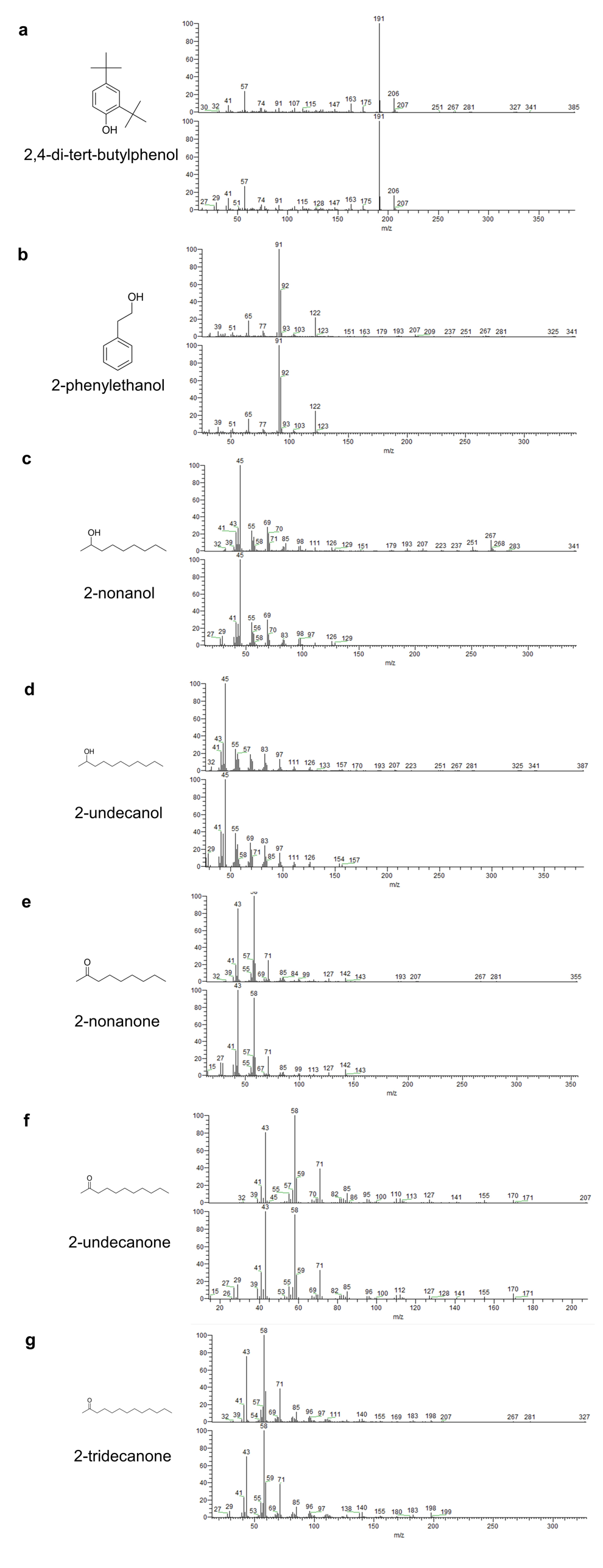

Supplement: Supplementary file 1 [file toxins-17-00449-s001.zip › Appendix figure S4.jpg]

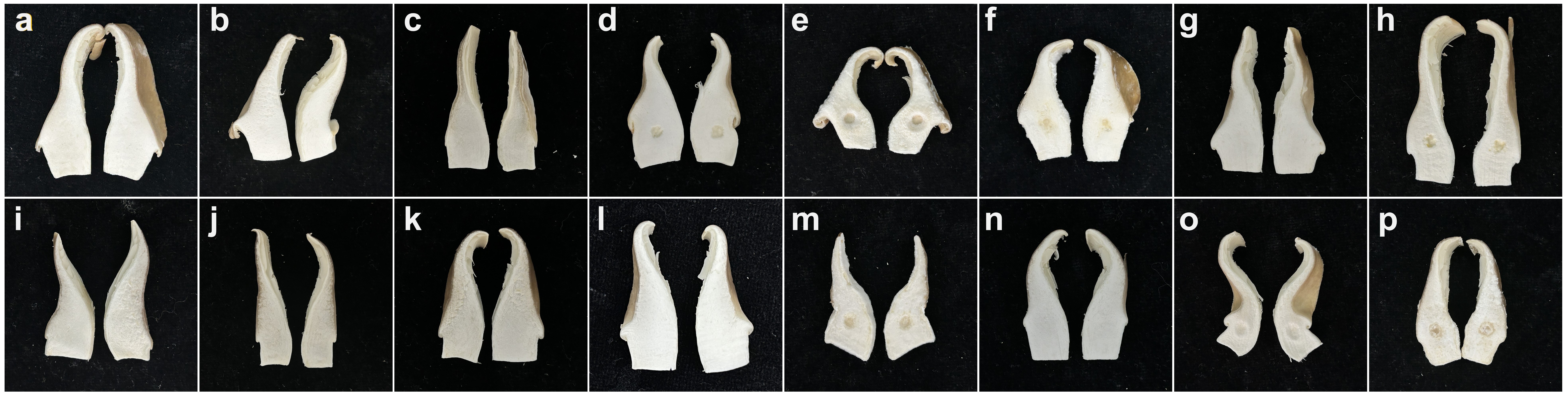

Supplement: Supplementary file 1 [file toxins-17-00449-s001.zip › Appendix figure S5.jpg]

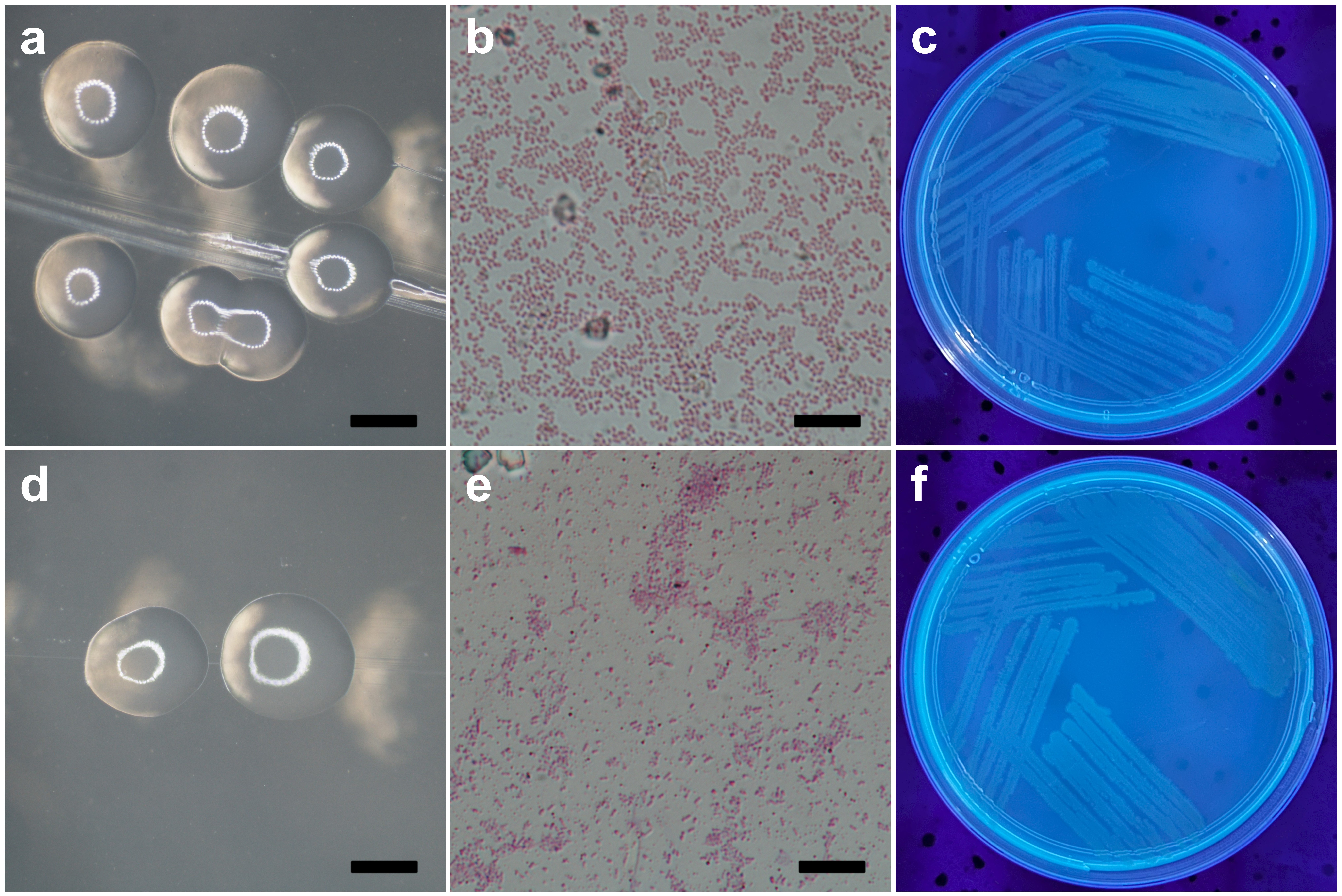

Supplement: Supplementary file 1 [file toxins-17-00449-s001.zip › Appendix Figure S1.jpg]
